# Correlation of methane production with physiological traits in *Trichodesmium* IMS 101 grown with methylphosphonate at different temperatures

**DOI:** 10.3389/fmicb.2024.1396369

**Published:** 2024-06-04

**Authors:** Chuze Zou, Xiangqi Yi, He Li, Mina Bizic, Ilana Berman-Frank, Kunshan Gao

**Affiliations:** ^1^State Key Laboratory of Marine Environmental Science, College of the Ocean and Earth Sciences, Xiamen University, Xiamen, China; ^2^Polar and Marine Research Institute, College of Harbor and Coastal Engineering, Jimei University, Xiamen, China; ^3^Co-Innovation Center of Jiangsu Marine Bio-industry Technology, Jiangsu Ocean University, Lianyungang, China; ^4^Department of Environmental Microbiomics, Institute of Environmental Technology, Technical University of Berlin, Berlin, Germany; ^5^Department of Plankton and Microbial Ecology, Leibniz Institute of Freshwater Ecology and Inland Fisheries (IGB), Stechlin, Germany; ^6^Department of Marine Biology, Leon H. Charney School of Marine Sciences, University of Haifa, Haifa, Israel

**Keywords:** cyanobacteria, diazotroph, growth, methane, N_2_-fixation, photosynthesis, phosphorus, *Trichodesmium*

## Abstract

The diazotrophic cyanobacterium *Trichodesmium* has been recognized as a potentially significant contributor to aerobic methane generation via several mechanisms including the utilization of methylphophonate (MPn) as a source of phosphorus. Currently, there is no information about how environmental factors regulate methane production by *Trichodesmium*. Here, we grew *Trichodesmium* IMS101 at five temperatures ranging from 16 to 31°C, and found that its methane production rates increased with rising temperatures to peak (1.028 ± 0.040 nmol CH_4_ μmol POC^−1^ day^−1^) at 27°C, and then declined. Its specific growth rate changed from 0.03 ± 0.01 d^−1^ to 0.34 ± 0.02 d^−1^, with the optimal growth temperature identified between 27 and 31°C. Within the tested temperature range the Q_10_ for the methane production rate was 4.6 ± 0.7, indicating a high sensitivity to thermal changes. In parallel, the methane production rates showed robust positive correlations with the assimilation rates of carbon, nitrogen, and phosphorus, resulting in the methane production quotients (molar ratio of carbon, nitrogen, or phosphorus assimilated to methane produced) of 227–494 for carbon, 40–128 for nitrogen, and 1.8–3.4 for phosphorus within the tested temperature range. Based on the experimental data, we estimated that the methane released from *Trichodesmium* can offset about 1% of its CO_2_ mitigation effects.

## 1 Introduction

Methane (CH_4_ ) is a crucial part of the carbon cycle and a potent greenhouse gas, with a global warming potential of more than 80 times that of CO_2_ over a 20-year period (IPCC, 2013). Intriguingly, methane is typically supersaturated in the ocean’s top mixed layer (Lamontagne et al., 1973; Scranton and Brewer, 1977; Weber et al., 2019), a phenomenon known as “the marine methane paradox.” The paradoxical nature of this phenomenon stems from the historically prevailing recognition that biogenic methane is produced exclusively by methanogenic archaea in a strictly anoxic environment, a condition incompatible with the oxic state of ocean top mixed layer (Karl and Tilbrook, 1994).

Recent studies show that phytoplankton, including cyanobacteria, can release methane in both oceanic and fresh surface waters (Karl et al., 2008; Bižić et al., 2020; Günthel et al., 2020; Klintzsch et al., 2020; Liu et al., 2022; Mao et al., 2022; Klintzsch et al., 2023). Precursors for aerobic CH_4_ production include various methylated substances, such as C-P bond methylphosphonate (MPn) (Karl et al., 2008), C-N bond trimethylamine (Bižić et al., 2018), monomethylamine, and glycine betaine (Wang et al., 2021), as well as C-S bound methylsulfonyl propionate and methionine (Damm et al., 2010; Lenhart et al., 2016; Klintzsch et al., 2019). In addition, other phytoplankton groups, such as *Chrysochromulina* sp., *Emiliania huxleyi*, *Navicula* sp., and *Leptocylindrus danicus* (Günthel et al., 2020; Klintzsch et al., 2020, 2023), can also produce methane through photosynthesis-linked pathways that are yet to be explored. A notable source of CH_4_ comes from the lysis of MPn C-P bond by specific microbes and cyanobacteria (Karl et al., 2008; Martinez et al., 2013; Repeta et al., 2016; Taenzer et al., 2020), making them primary contributors to methane production in the oceans.

The aerobic metabolism of MPn serves as a crucial source of phosphorus and subsequently a significant source of methane (Karl et al., 2008; Repeta et al., 2016; Von Arx et al., 2023). Despite dissolved inorganic phosphate (DIP) typically being the most bioavailable form of phosphorus, its availability is commonly limited in pelagic surface waters (Sañudo-Wilhelmy et al., 2001; Dyhrman et al., 2002). It has been documented that concentrations of dissolved organic phosphorus (DOP) in the open oceans are often much higher than those of DIP (Björkman and Karl, 2003; Karl and Björkman, 2015). The primary sources of DOP in the ocean originate from biological processes that include exudation, viral lysis, autolysis and cell death, and grazing (Karl and Björkman, 2015). C-P bond phosphonates are broadly distributed in the ocean, with the nuclear magnetic resonance (NMR) spectra of ultrafiltered dissolved organic matter (DOM) revealing that phosphonates (21%) are the second predominant components, after phosphate esters (73%), of high molecular weight dissolved organic phosphorus (HMWDOP) in the Pacific Ocean, the Atlantic Ocean, and the North Sea (Clark et al., 1998; Kolowith et al., 2001; Repeta et al., 2016). At station ALOHA, MPn and its precursor 2-hydroxyethylphosphonate (2-HEP) account for approximately 20% of the HMWDOP (Repeta et al., 2016). MPn can be synthesized by microbes, such as the archaeon *Nitrosopumilus maritimus* (Metcalf et al., 2012) and by other abundant marine bacteria, such as *Candidatus* Pelagibacter sp. (Born et al., 2017). The catabolism of MPn involves active transport into the cytoplasm through the phosphonate-specific ABC transporter system integrated by the phnCDE complex (Stasi et al., 2019) and subsequent degradation by the protein complex phnGHIJK (Kamat et al., 2011; Amstrup et al., 2023).

*Trichodesmium*, a prominent N_2_-fixing organisms in the pelagic oceans, has long been recognized as a primary contributor to oceanic N_2_ fixation (Capone et al., 1997; Bergman et al., 2013), supplying the “new” nitrogen within the euphotic zones of the tropical and subtropical regimes (Zehr and Capone, 2020). N_2_ fixation and growth of *Trichodesmium* in the ocean is often constrained by the availability of inorganic phosphorus (Pi) (Sañudo-Wilhelmy et al., 2001; Frischkorn et al., 2018; Wang, 2022). Under Pi-limiting conditions, *Trichodesmium* exhibits weak competitiveness for Pi due to its lower specific affinities compared to other phytoplankton (Mccarthy and Carpenter, 1979; Sohm and Capone, 2006; Dyhrman, 2016). However, it can effectively utilize phosphate esters as a dependable source of phosphorus, supported by high levels of alkaline phosphatase (APase) activity (Mccarthy and Carpenter, 1979; Stihl et al., 2001; Sohm and Capone, 2006). Furthermore, *Trichodesmium* acquires phosphorus from phosphonate compounds, including MPn, through the C-P lyase pathway (Dyhrman et al., 2006), which leads to methane production as a byproduct (Karl et al., 2008). Hence, *Trichodesmium* is significant not only as a contributor to new nitrogen input for ocean but also as a potential source of ocean methane. However, little has been documented on the impacts of environmental drivers (e.g., temperature, P-availability) on methane production by *Trichodesmium*.

*Trichodesmium*’s temperature tolerance ranges from18°C to 34°C when grown on the ingoranic phosphate, with optimal temperatures between 26–28°C (Breitbarth et al., 2007; Chappell and Webb, 2010; Fu et al., 2014). Rising temperatures impact diverse physiological and biochemical processes, including nitrogen fixation, respiration, carbon fixation, and growth (Breitbarth et al., 2007; Yvon-Durocher et al., 2012; Fu et al., 2014). As ocean warming intensifies, thermal stratification in the upper ocean stabilizes, reducing upward supply of dissolved inorganic phosphate (DIP) (Behrenfeld et al., 2006; Polovina et al., 2008; Somavilla et al., 2017; Ulloa et al., 2019). This may promote *Trichodesmium* to utilize more MPn, which may boost methane emission. Nevertheless, the thermal response of methane production by *Trichodesmium* has not been examined thus far. We hypothesize that changes in temperatures would alter levels of methane production by *Trichodesmium* grown on Mpn, since its utilization should correlate with the temperature-dependent assimilations of carbon, nitrogen, and phosphorus. To test this hypothesis, we employed MPn as the phosphorus source, grew *Trichodesmium* under various temperatures ranging from 16 to 31°C, and examined the correlations between methane production and several pivotal physiological processes, including assimilation of carbon, nitrogen, and phosphorus, as well as the specific growth rates. We found that changes in temperature affect the methane production of *Trichodesmium* grown on Mpn in parallel with the rates of C/N/P assimilations and growth.

## 2 Materials and methods

### 2.1 Culture conditions

*Trichodesmium* IMS101 stock culture was grown in nitrogen-free YBCII medium (Chen et al., 1996) containing 5 μM methylphosphonate (MPn, Aladdin, CAS 993-13-5, ≥98%). The stock culture was kept at 27°C, and the light intensity was set at 110 μmol photons m^−2^ s^−1^ with a light–dark cycle of 12:12 h (light source: white LED tubes; light period: 08:00 to 20:00 local time). The cultures were run at five different temperatures (16, 20, 23, 27, 31°C), with all other environmental conditions maintained identical to those of the stock cultures. No other P source or dissolved inorganic phosphorus (DIP) was intentionally added to the cultures. The experimental cultures were kept in the exponential growth phase through regular dilutions performed every 3–15 days depending on the temperatures and growth rates. The semi-continuous cultures ensured that chlorophyll-*a* (Chl-*a*) concentrations consistently fell within the range of 0.005–0.05 μg mL^−1^. The experimental cultures were acclimated to their respective temperatures for more than 6 months prior to measuring physiological and biochemical parameters.

### 2.2 Chl-*a* and specific growth rate

Chl-*a* concentration was determined using the spectrophotometric method. The cells were filtered onto GF/F filters and subsequently extracted in pure methanol overnight at 4°C in the dark. After extraction, the samples were centrifuged at 12,000 g for 4 min. The resulting supernatant was then scanned for absorbance across the wavelength range of 250–800 nm using a spectrophotometer (Cary 60, Agilent, CA, United States). Chl-*a* concentration was calculated using the following formula (Ritchie, 2006):


$$Chl\;a\;\left(\mathrm{\mu g}\;{\mathrm{mL}}^{-1}\right)\;=12.9447\times \left({OD}_{665}-{OD}_{750}\right)\text{,}$$

where OD_665_ and OD_750_ were absorbance at wavelengths 665 nm and 750 nm, respectively. The following equation was used to calculate the specific growth rate $\left(\mu \right)$ based on the Chl-*a* concentration:


$$\mu \;\left(d^{-1}\right)=\frac{\ln\;m_{2}-\ln\;m_{1}}{t_{2}-t_{1}}\text{,}$$

where *m*_2_ and *m*_1_ are the Chl-*a* values at time *t*_2_ and *t*_1_, respectively.

The following equation modified by Norberg (2004) was utilized to fit the thermal growth curve of *Trichodesmium*:


$$\mu \left(T\right)={ae}^{bT}\left[1-{\left(\frac{T-z}{W}\right)}^{2}\right]\text{,}$$

where the specific growth rate (μ) is a function of temperature (T). In this equation, the coefficient w represents the thermal niche width, whereas the explicit biological significance of coefficients a, b, and z remain unspecific. Collectively, these four coefficients can be used to derive both the maximum growth rate and the optimum growth temperature (T_opt_):


$$T_{\mathrm{opt}}=\frac{bz-1+\sqrt{w^{2}b^{2}+1}}{b}\text{,}$$

### 2.3 Chl-*a* fluorescence

The effective photosynthetic quantum yield of photosystem II (Φ_II_) and relative electron transport rate (rETR) were measured by Multi-color PAM (Multi-color PAM, Walz). Samples were acclimated to white actinic light with photon flux intensities similar to the growth conditions for 2 min to measure F_s_, and then F_m_′ was measured using a saturating pulse (8,000 μmol photons m^−2^ s^−1^, 0.8 s) to obtain the effective quantum yield (Φ_II_) as follows (Genty et al., 1989):


$$\Phi_{II}=\left({F_{m}}^{\prime }-F_{s}\right)/{F_{m}}^{\prime }\text{.}$$

Subsequently, rapid light curves (RLC) were measured at 11 actinic light levels [E], from 5 to 2,904 μmol photons m^−2^ s^−1^ with each light exposure lasting for 30 s. Relative electron transport rates (rETR) were calculated as follows (Ralph and Gademann, 2005):


$$\mathrm{rETR}=E\times \Phi_{II}$$

The RLC was fitted by the following equation (Supplementary Figure S1) (Eilers and Peeters, 1988),


$$\mathrm{rETR}=\frac{E}{a\times E^{2}+b\times E+c}\text{,}$$

where a, b, and c are the fitting coefficients. These three coefficients were used to derive the photosynthetic light-harvesting efficiency (α), maximum relative electron transport rate (rETR_max_):


α=1/c,

$${\mathrm{rETR}}_{\text{max}}=1/\left(b+2\times \sqrt{a\times c}\right)\text{.}$$

### 2.4 Methane production

A Cavity Ring-Down Spectroscopy gas analyzer (Picarro Model G2308, CA, United States) was used to measure methane concentrations. A 350 mL sample (*V_l_*) was placed within a polycarbonate (PC) bottle, leaving a 270 mL headspace (*V_g_*). The PC bottle was sealed using a silicone stopper to ensure an airtight condition. The silicone stopper was equipped with two three-way valves for sampling. During the incubation of the samples at each of the temperatures (16, 20, 23, 27, 31°C) for methane measurement, 200 mL of gas in the bottle was replaced with sterile air every 12 h. The methane content in the headspace gas was shaken to equilibrate the dissolved gas with the headspace before subsequent measurement using the Picarro analyzer according to Lenhart et al. (2016). The total methane production rates ( $b_{{CH}_{4}}$ ) were calculated based on the modified formula (Johnson et al., 1990):


$$C_{g}=\left(C_{M}\times 470-C_{\mathrm{air}}\times 200\right)/270\text{,}$$

$$\begin{array}{l} b_{{CH}_{4}}\;\left(\mathrm{nmol}\;{\mathrm{day}}^{-1}\right)=\left(C_{g2}-C_{g1}\right)\times \left(\beta /22.356\times RT+V_{g}/V_{l}\right)\times \\ \;V_{l}/\Delta t\text{,} \end{array}$$

where *C_g_* indicates the methane concentration in the headspace (nmol L^−1^), *C_M_* represents the methane concentration in the headspace after replacing with the sterile air, *C*_air_ denotes the methane concentration of the sterile air, and *C*_*g*1_ and *C*_*g*2_ signify the methane concentrations at two time points, *t*_1_ and *t*_2_. Additionally, β is the Benson coefficient. *V_g_* represents the headspace volume, while *V_l_* corresponds to the volume of the culture medium. Methane production rates specific to *Trichodesmium* were determined based on the rates obtained from samples extracted from fractions containing heterotrophic bacteria. Several control experiments were conducted to assess the potential influence of heterotrophic bacteria and to account for systematic errors. To test the bacterial contribution to methane production we filtered YBCII media through a 1.2 μm polycarbonate membrane, which lacked *Trichodesmium* but retained the heterotrophic bacteria in the cultures. It should be noted that the bacteria attached to the filaments of *Trichodesmium* might not be filtered off into the medium.

### 2.5 Carbon and nitrogen assimilation

In this study carbon and nitrogen assimilation rates were determined by assessing changes over time for particulate organic carbon (POC) and particulate organic nitrogen (PON) respectively. Briefly, samples for POC and PON measurements were taken at 0, 12, and 24 h after the start of the light period. The changes in particulate organic nitrogen (PON) and particulate organic carbon (POC) were then analyzed to determine the assimilation rate of carbon and nitrogen. Upon sampling, cells were filtered onto pre-combusted (450°C, 4 h) GF/F filters and rinsed with 100 mL fresh nitrogen-free YBCII. Subsequently, the filters were acidified for 24 h in HCl fumes and then dried for 24 h to remove unassimilated inorganic carbon. An elemental analyzer (Vario EL cube, Elementary, Germany) was used to quantify POC and PON. Changes in POC or PON between samples taken at 12 h and 0 h represented the assimilation during the light period, and changes between samples taken at 24 h and 0 h represented the daily assimilation. The results were comparable to the POC and PON production rates, which were calculated as POC or PON content (nmol Chl *a*^−1^) × specific growth rate μ (d^−1^, Supplementary Figure S2) (Tong et al., 2019).

We acknowledge that the POC changes effectively represent integrated assimilation of inorganic carbon and recycling of organic carbon leaked into the media by *Trichodesmium*. Similarly, the nitrogen assimilation rates, as determined in this study, are indicative of the sum of both N_2_-fixation and the recycling of biogenic nitrogen leaked into the media by *Trichodesmium*.

### 2.6 Dissolved and particulate phosphorus measurement

To determine dissolved inorganic phosphorus (DIP), the samples were filtered through a 0.22 μm cellulose acetate membrane and were then analyzed using an auto-analyzer (AA3, Seal, Germany) at room temperature. For the measurement of particulate phosphorus (PP), we followed the Solórzano method (Solórzano and Sharp, 1980). In brief, the samples were filtered on pre-combusted (450°C, 4 h) GF/F filters (25 mm, Whatman, United States), and rinsed with 100 mL phosphorus-free YBCII artificial seawater. Subsequently, the filters were soaked with 0.017 M MgSO_4_, dried at 95°C, and then baked for 2 h at 450°C. Before measurement, the samples were hydrolyzed with acid (0.2 M HCl) at 80°C for 30 min. These procedures convert PP to DIP, which was subsequently quantified with an auto-analyzer (see above). The assimilation rates of phosphorus during a daily cycle were calculated using the PP contents at 0 h, 12 h, and 24 h.

### 2.7 Thermal dependence of metabolic processes

The thermal dependence of methane production and the related assimilation of carbon, nitrogen and phosphorus was analyzed using the Boltzmann-Arrhenius equation (Padfield et al., 2016):


$$\ln\left(b\left(T\right)\right)=E_{a}\left(\frac{1}{k\;T_{c}}-\frac{1}{k\;T}\right)+\ln\left(b\left(T_{c}\right)\right)\text{,}$$

where *b*(*T*) represents the metabolic rate at temperature T (Kelvin, K), *k* Boltzmann’s constant (8.62 × 10^−5^ eV K^−1^), *b*(*T_c_*) the rate of metabolism normalized to an arbitrary reference temperature, *T_c_* = 25°C, and *E_a_* is the activation energy (in electron volts, eV) for the metabolic process. Supra-optimal temperatures could deviate the metabolic rate from the Boltzmann-Arrhenius equation. Therefore, corresponding values, usually those observed at 31°C, were excluded from this analysis.

The sensitivity of methane production and assimilation of carbon, nitrogen and phosphorus to temperature changes (Q_10_) from 16°C to 27°C was assessed by the following model (Van't Hoff and Lehfeldt, 1899):


$$Q_{10}={\left(\frac{{\mathrm{Rate}}_{2}}{{\mathrm{Rate}}_{1}}\right)}^{\frac{10}{T_{2}-T_{1}}}\text{,}$$

where Rate_1_ and Rate_2_ indicate metabolic rates at 16°C (T_1_) and 27°C (T_2_), respectively.

### 2.8 Statistical analysis

The data were provided as the means of three replicates (independent cultures) with standard deviation (SD) (*n* = 3). To examine the statistical differences between treatments, one-way ANOVA and Tukey’s test were used. The Brown-Forsythe test and Shapiro–Wilk test were used to check data homoscedasticity and normality, respectively.

## 3 Results

### 3.1 Specific growth rates, ratio of Chl-*a* to POC, ratio of POC to PON and Chl-*a* fluorescence

**Figure 1:**
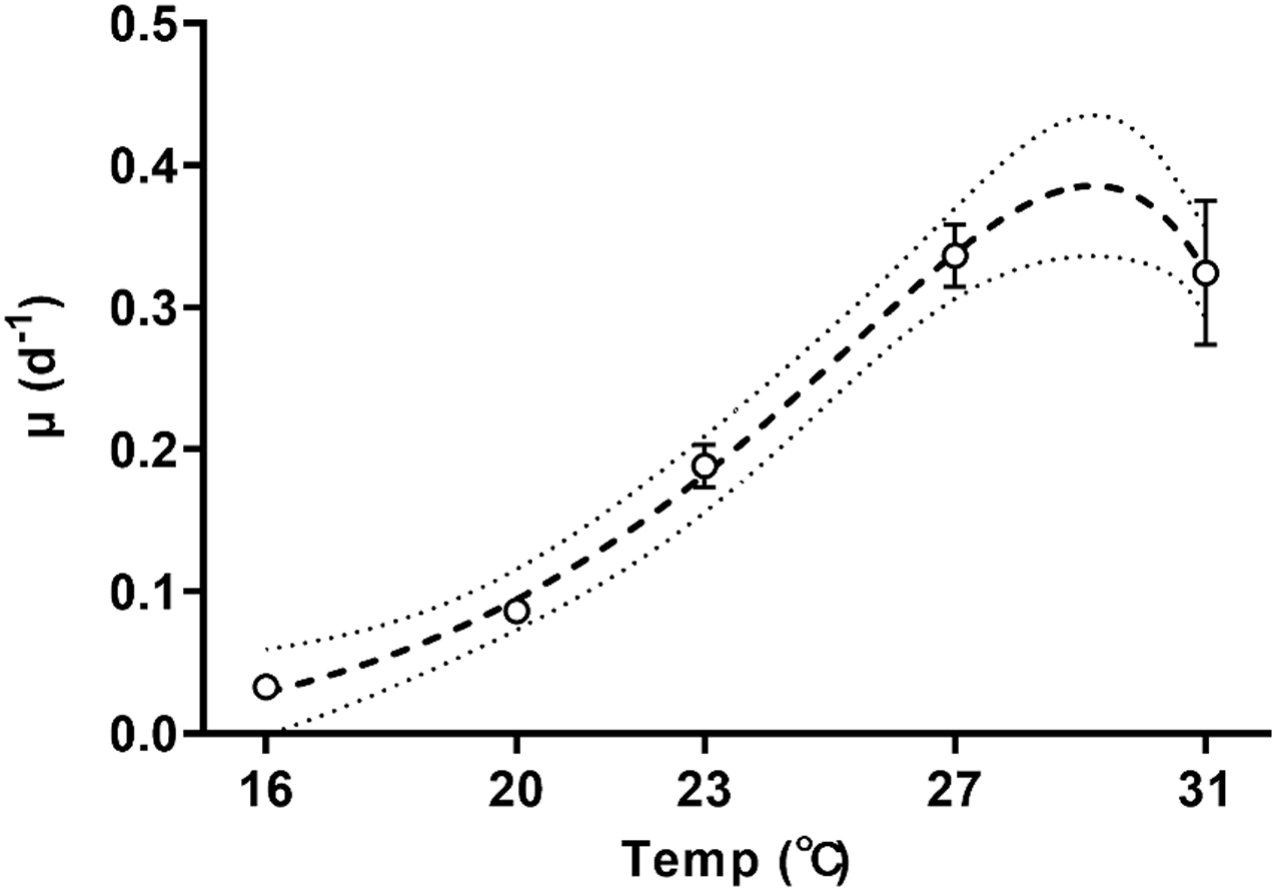
Specific growth rates of *Trichodesmium* IMS101 during the exponential stage after having acclimated at each of the specific temperatures [ranging from 16 to 31°C for 6 months]. The dotted line represents the 95% prediction bands of the best-fit line. Values represent the means ± SD of independent biological replicates (*n* = 3).

The specific growth rate of *Trichodesmium* increased with temperature (Figure 1; one-way ANOVA, *p* < 0.001) and was sensitive to temperature changes, with a Q_10_ value (the rate increase fold for every 10-degree rise in the temperature) of growth rate for temperatures ranged from 16°C to 27°C reaching 8.6 ± 2.3. As the culture temperature increased from 16°C to 27°C, the specific growth rate exhibited a tenfold increase from 0.03 ± 0.01 d^−1^ to 0.34 ± 0.02 d^−1^ (Figure 1, Tukey’s test, *p* = 0.0001). Increasing the growth temperature to 31°C did not significantly change the specific growth rate (0.32 ± 0.05 d^−1^, Figure 1, Tukey’s test, *p* = 0.999). According to the Norberg equation, the optimal growth temperature (*T*_opt_) was 29.2°C, corresponding to a maximum growth rate of 0.38 d^−1^.

The ratio of Chl-*a* to POC increased with the rise in temperatures (one-way ANOVA, *p* < 0.0001). The Chl *a*: POC was 68 ± 1 ng Chl *a* μmol POC^−1^ at the lowest growth temperature of 16°C, and increased by 100% to 136 ± 3 ng Chl *a* μmol POC^−1^ at 31°C (Table 1; Tukey’s test, *p* < 0.0001).

**Table 1:** Chlorophyll *a* content normalized to cellular particular organic carbon (Chl *a*: POC), ratio of POC to PON (POC: PON), effective quantum yield (Φ_II_), photosynthetic light-gathering efficiency (α), maximum relative electron transport rate (rETR_max_) of *Trichodesmium* IMS101 after having acclimated for 6 months to specific temperatures ranging from 16 to 31°C.

Growth Temp (°C)	Chl *a*: POC (ng: μmol)	POC: PON (mol: mol)	Φ_II_	α	rETR_max_ (μmol e m^−2^ s^−1^)
16	68 ± 1^a^	6.60 ± 0.24^ab^	0.17 ± 0.02^a^	0.22 ± 0.02^a^	73 ± 7^a^
20	101 ± 1^b^	6.23 ± 0.04^ac^	0.30 ± 0.02^b^	0.46 ± 0.02^b^	135 ± 10^b^
23	78 ± 13^ac^	7.00 ± 0.38^b^	0.27 ± 0.02^b^	0.35 ± 0.03^b^	127 ± 9^b^
27	120 ± 6^d^	6.12 ± 0.14^ac^	0.49 ± 0.01^c^	0.63 ± 0.01^c^	228 ± 4^c^
31	136 ± 3^d^	5.64 ± 0.26^c^	0.49 ± 0.01^c^	0.61 ± 0.01^c^	224 ± 5^c^

The rETR_max_ and α were derived from the rapid light curves (Supplementary Figure S1). Values represent the means ± SD of independent biological replicates (*n* = 3).

In contrast, the ratios of POC to PON were negatively correlated with temperatures (Table 1; one-way ANOVA, *p* = 0.0004). When the growth temperature increased from 16°C to 31°C, the ratios of POC to PON decreased by 15% from 6.60 ± 0.24 to 5.64 ± 0.26 (Table 1; Tukey’s test, *p* = 0.004).

The effective quantum yield (Φ_II_), a measure indicative of the photosynthetic efficiency of *Trichodesmium*, increased with rising temperature (one-way ANOVA, p < 0.0001). The value of Φ_II_ was 0.17 ± 0.02 at the lowest temperature of 16°C and increased by 188% to 0.49 ± 0.01 (Table 1; Tukey’s test, *p* < 0.0001) at 31°C. The relative maximum electron transport rate (rETR_max_) and photosynthetic light-use efficiency (α) showed a similar pattern to Φ_II_. Specifically, rETR_max_ increased by 207% from 73 ± 7 to 224 ± 4 μmol e m^−2^ s^−1^ (Table 1, one-way ANOVA, *p* < 0.0001), while the value of α increased by 177% from 0.22 ± 0.02 to 0.61 ± 0.01 (Table 1, one-way ANOVA, *p* < 0.0001) with the temperature increasing from 16 to 31°C.

### 3.2 Methane production

**Figure 2:**
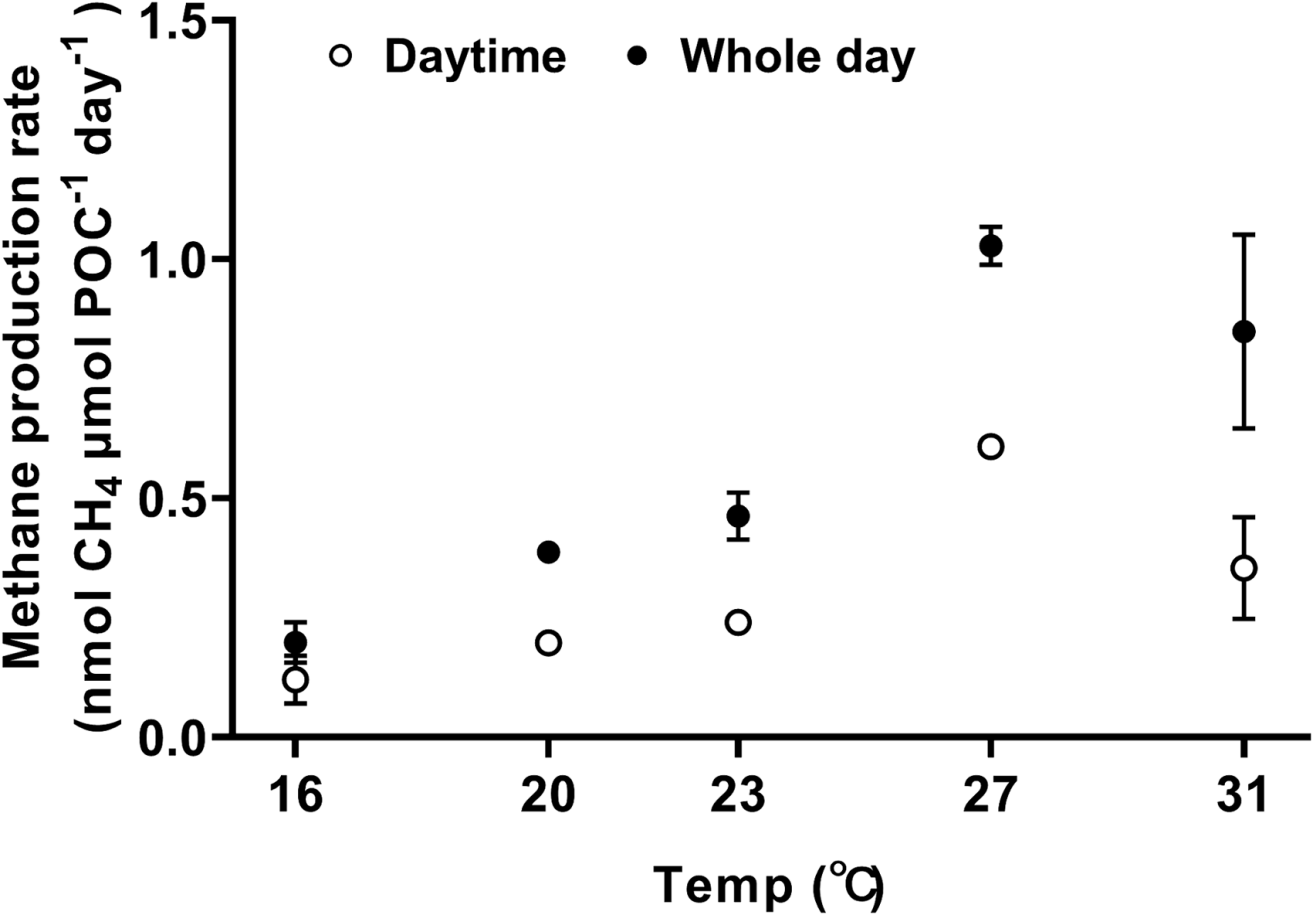
The effect of growth temperature on methane production by *Trichodesmium* IMS101. The cultures were acclimated to the temperatures for 6 months, and the methane production rates were normalized to particulate organic carbon (POC). The hollow circles represent methane production during the daytime (12 h), whereas the solid ones represent the diel production, summing that of light and dark periods. Values represent the means ± SD of independent biological replicates (*n* = 3).

Methane was produced during both the day and the night, exhibiting a typical temperature response curve (Figure 2, one-way ANOVA, *p* < 0.0001) and demonstrated sensitivity to temperature changes, with a Q_10_ value for temperature ranged from 16°C to 27°C, reaching 4.6 ± 0.7. At 16°C, the methane production rates during the light period and daily cycle were 0.120 ± 0.050 nmol CH_4_ μm POC^−1^ day^−1^ and 0.198 ± 0.042 nmol CH_4_ μmol POC^−1^ day^−1^, respectively (Figure 2). When the growth temperature increased to 27°C, the rate of methane production reached the maximum, with light-period and daily-cycle values increasing by 407% to 0.608 ± 0.018 nmol CH_4_ μmol POC^−1^ day^−1^ (Tukey’s test, *p* < 0.0001) and by 419% to 1.028 ± 0.040 nmol CH_4_ μmol POC^−1^ day^−1^ (Tukey’s test, *p* < 0.0001), respectively (Figure 2). The activation energy (*E_a_*) for methane production was 1.09 ± 0.13 eV for light period and 1.08 ± 0.08 eV for daily-cycle. Notably, the amount of methane production in each treatment increased with incubation time (Supplementary Figure S3A) and showed a positive correlation with biomass (Supplementary Figure S4). Methane production was also observed in bacterial controls, and the production rate increased with rising temperature (Supplementary Figure S3B). *Trichodesmium* dominated the methane production, with heterotrophic bacterial contribution to the total production being 19.7% at 16°C, 1.5% at 20°C, 3.8% at 23°C, 1.9% at 27°C, and 11.4% at 31°C, respectively (Supplementary Figure S3A). Methane production by *Trichodesmium* was derived by subtracting the bacterial production from the total production, with the possible contribution of the attached heterotrophic bacteria to the filaments of *Trichodesmium* being ignored.

### 3.3 Carbon, nitrogen, and phosphorus assimilation

**Figure 3:**
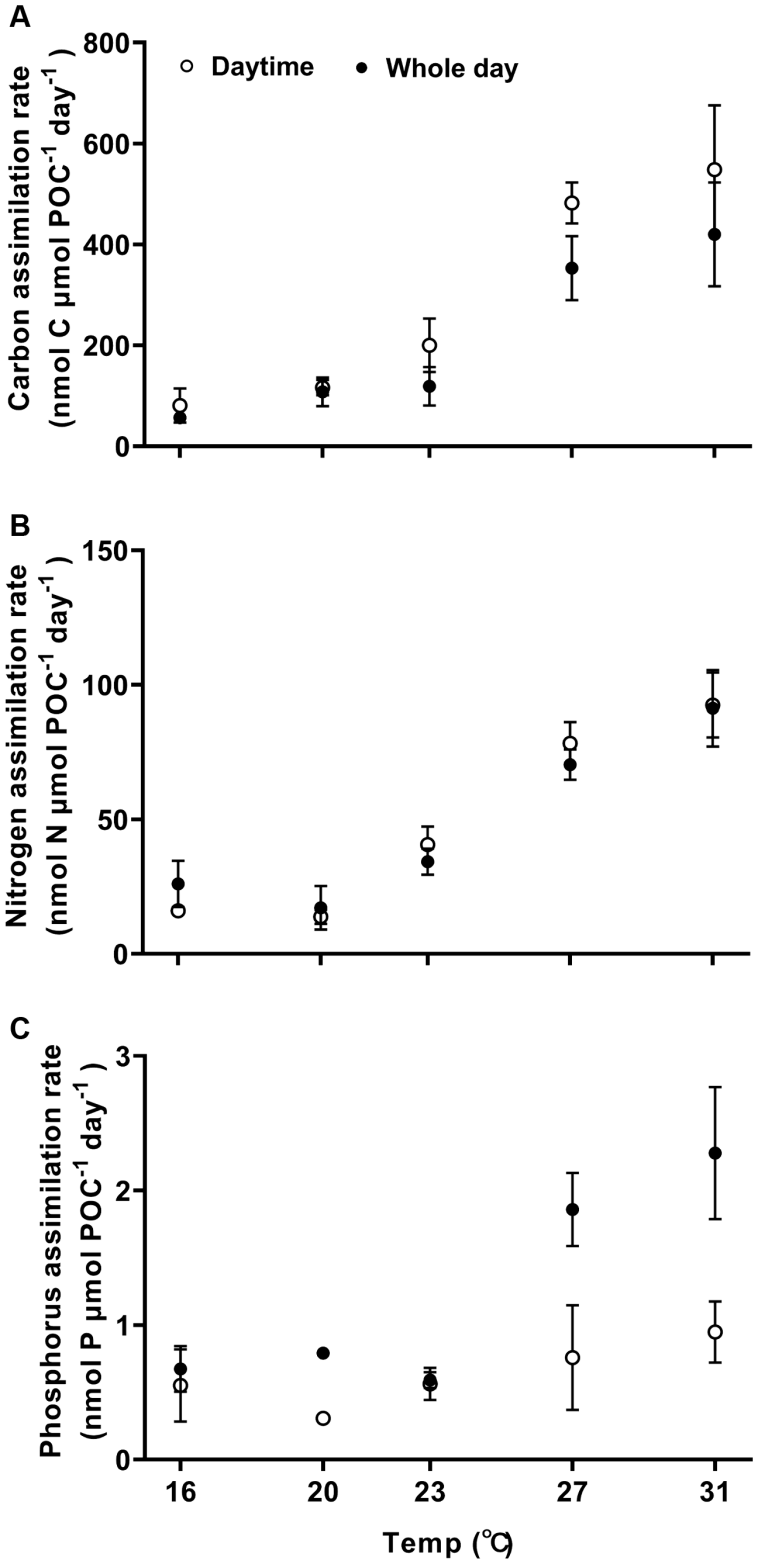
Carbon, nitrogen, and phosphorus assimilation rates **(A–C)** of *Trichodesmuim* IMS 101 normalized to particular organic carbon (POC) during the light period (hollow circle) or the diel cycle (solid circle) after being acclimated for 6 months to specific temperatures. Values represent the means ± SD of independent biological replicates (*n* = 3).

Carbon, nitrogen, and phosphorus assimilation rates increased with higher growth temperatures (Figure 3, one-way ANOVA, *p* < 0.0001, *p* < 0.0001, and *p* < 0.0001), demonstrating a high sensitivity to temperature changes. The Q_10_ values for temperature ranged from 16°C to 27°C for POC, PON and POP production were 4.9 ± 0.7, 2.6 ± 0.5, and 2.6 ± 0.3, respectively. When the temperature was raised from 16°C to 31°C, the light-period and daily rates of carbon assimilation increased by 577% from 81 ± 34 to 548 ± 128 nmol C nmol POC^−1^ day^−1^ (Tukey’s test, *p* < 0.0001) and by 650% from 56 ± 11 to 420 ± 103 nmol C μmol POC^−1^ day^−1^ (Tukey’s test, *p* = 0.0002), respectively (Figure 3A). The activation energy (*E_a_*) for carbon assimilation was 1.26 ± 0.15 eV for light period and 1.19 ± 0.19 eV for daily-cycle. As the growth temperature increased from 16°C to 31°C, the nitrogen assimilation rate increased by 475% from 16 ± 2 to 92 ± 12 nmol N μmol POC^−1^ day^−1^ (Tukey’s test, *p* < 0.0001) during the light period, and increased by 250% from 26 ± 6 to 91 ± 14 nmol N μmol POC^−1^ day^−1^ (Tukey’s test, *p* < 0.0001) (Figure 3B). The activation energy (Ea) for nitrogen assimilation was 1.19 ± 019 eV for light period and 0.78 ± 0.25 eV for daily-cycle. The phosphorous assimilation rate was 0.7 ± 0.2 nmol P μmol POC^−1^ day^−1^ for the light–dark period at 16°C, and increased by 228% from to 2.3 ± 0.5 nmol P μmol POC^−1^ day^−1^ at 31°C (Figure 3C; Tukey’s test, *p* < 0.0001).

**Figure 4:**
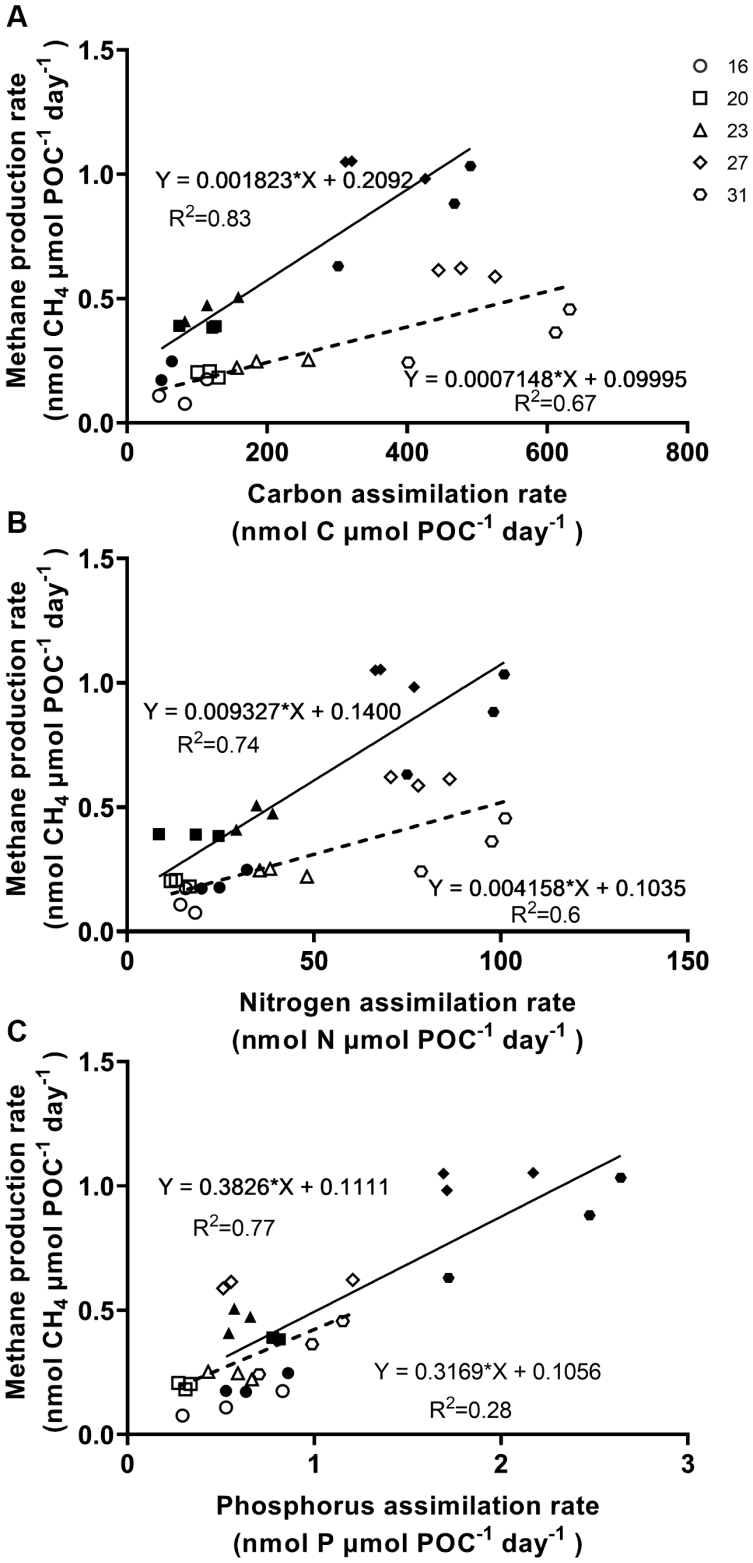
Methane production rates of *Trichodesmium* IMS101 correlated with assimilation rates of carbon **(A)**, nitrogen **(B)**, and phosphorus **(C)**, which were derived from Figures 2, 3. The hollow symbols and dotted line represent the correlations for the light period, while the solid symbols and lines represent the daily values. The numbers next to the different symbols indicate the growth temperatures (°C). Each data point represents an independent biological replicate, and different symbols represent different biological replicates.

**Figure 5:**
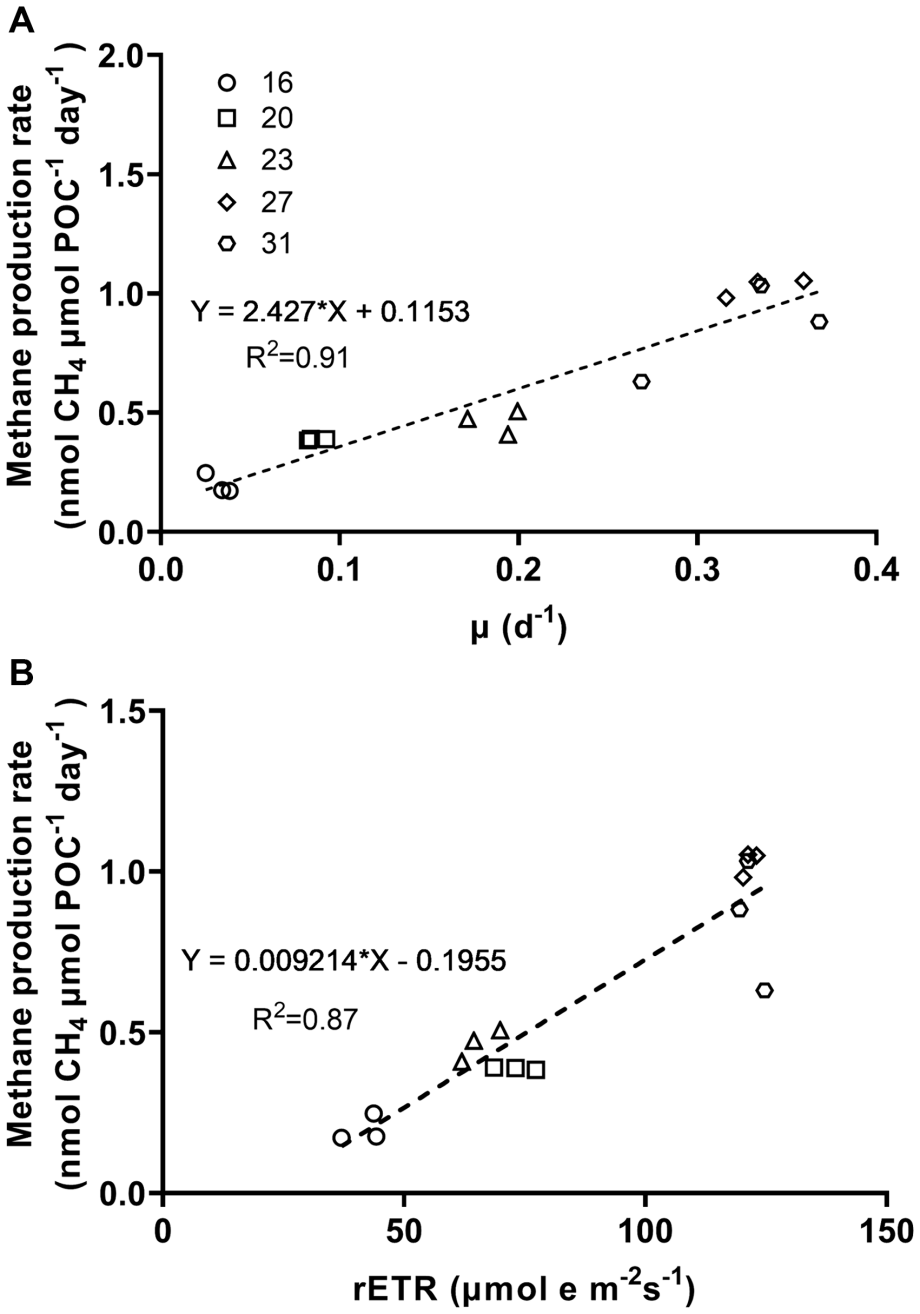
Methane production rates of *Trichodesmium* IMS101 as a function of specific growth rates **(A)** and relative electron transport rates (rETR) **(B)**. The rETR, were derived from the rapid light curves (Supplementary Figure S1), and represent rates measured at the acclimation light level. The correlation of methane production and the specific growth rates was based on Figures 1, 2. The numbers next to the symbols indicate the growth-acclimation temperatures (°C). Each data point represents an independent biological replicate, and different symbols represent different biological replicates.

The methane production correlated positively with rates of C, N, and P assimilation, specific growth and relative electron transfer (Figures 4, 5). The daily methane production increased with higher carbon, nitrogen, and phosphorus assimilation (Figure 4). The correlation coefficients between the daily methane production and the assimilation of carbon, nitrogen and phosphorus were 0.83, 0.74, and 0.77, respectively (Figure 4, *p* < 0.0001, *p* < 0.0001, *p* < 0.0001). The methane production quotients (MPQ), ratio of the carbon, nitrogen or phosphorus assimilation rates to the methane production rates, were 253–494 for carbon, 40–128 for nitrogen, and 1.3–3.4 for phosphorus, respectively (Table 2).

**Table 2:** The quotients of carbon (C_as_: CH_4pro_), nitrogen (N_as_: CH_4pro_) and phosphorus (P_as_: CH_4pro_) assimilations to methane production rate, and the ratio of specific growth rate (μ: CH_4pro_) and relative electron transfer rate (rETR: CH_4pro_) to methane production rate in *Trichodesmium* IMS101 after having acclimated for 6 months to specific temperatures ranging from 16 to 31°C.

Growth Temp (°C)	C_as_: CH_4pro_	N_as_: CH_4pro_	P_as_: CH_4pro_	μ: CH_4pro_	rETR: CH_4pro_
16	227 ± 18^a^	128 ± 12^a^	3.39 ± 0.35^a^	0.17 ± 0.06^a^	215 ± 37^a^
20	278 ± 75^a^	40 ± 21^b^	2.04 ± 0.07^b^	0.22 ± 0.01^ac^	189 ± 13^ab^
23	253 ± 57^a^	74 ± 7.3^b^	1.28 ± 0.14^c^	0.41 ± 0.06^b^	142 ± 8^b^
27	345 ± 77^ab^	69 ± 82^b^	1.81 ± 0.23^bc^	0.33 ± 0.01^bc^	118 ± 4^b^
31	494 ± 31 ^b^	109 ± 11^ac^	2.70 ± 0.13^d^	0.39 ± 0.06^b^	150 ± 42^ab^

Values represent the means ± SD of independent biological replicates (*n* = 3).

The relationship between metabolic activity and growth rates to methane production rates, which was established based on the positive correlations of methane production with the specific growth rate (μ) and relative electron transport rate (rETR), provided the correlation coefficients (R-square) of 0.91 and 0.87, respectively (Figure 5, *p* < 0.0001, *p* < 0.0001). Under different growth temperatures, the ratio of specific growth rate to methane production rate ranged from 0.17 to 0.41 and that of rETR to methane production rate ranged from 118 to 215 (Table 2).

## 4 Discussion

The thermal dependence of key metabolic processes, including N_2_ fixation, photosynthetic CO_2_ fixation and specific growth rate, have been widely explored in *Trichodesmium* (Mulholland and Bernhardt, 2005; Breitbarth et al., 2007; Boatman et al., 2017). These studies have contributed to our understanding of its current distribution in natural environments and provided insights to predicting its future behavior under influence of climate change (Breitbarth et al., 2007; Fu et al., 2014; Jiang et al., 2018; Yi et al., 2020). This study presents the first report on how temperature impacts methane production in *Trichodesmium* and the relationship of methane production to the assimilation of carbon, nitrogen and phosphorus, although it has been known to release methane for almost two decades (Karl et al., 2008; Beversdorf et al., 2010; Repeta et al., 2016). Within the experimental temperature range of 16 to 31°C, the daily methane production increased with growth temperature and saturated at 27°C, with a corresponding value of 1.028 ± 0.040 nmol CH_4_ μmol POC^−1^ day^−1^ (Figure 2). Obvious linear positive correlations were detected between methane production and the assimilations of carbon, nitrogen and phosphorus (Figure 4).

Unlike the assimilation of carbon and nitrogen, which predominantly occurred during the light period (Figure 3), substantial methane production and phosphorus assimilation were observed during dark periods as well (Figure 2). In *Trichodesmium*, photosynthetic CO_2_ fixation and biological N_2_ fixation provide the substrates for other physiological and biochemical processes (Berman-Frank, 2001; Milligan, 2007). Moreover, the circadian clock of *Trichodesmium* confines the expression of genes related to CO_2_ and N_2_ fixation to light period (Ohki et al., 1992; Chen et al., 1996, 1998; Dodd et al., 2005; Masotti et al., 2007; Haydon et al., 2013). For MPn uptake and C-P cleavage, the required energy is directly provided by ATP (Kamat et al., 2011; Stasi et al., 2019). During the night, mitochondrial respiration likely served as the primary source of ATP, which consumes the organic carbon stored during the daytime period (Figure 3A). Within the temperature range of 16–31°C, the specific growth, the methane production rate and the assimilation rates of carbon, nitrogen and phosphorus exhibited high sensitivity to thermal changes with Q10 greater than 2 (Supplementary Table S1). The methane production of *Trichodesmium* saturated at 27°C and decreased when the temperature exceeded 31°C, indicating a threshold for warming to promote methane production. Methane production in the associated heterotrophic bacteria (control) also increased with rising temperatures and peaked at 31°C (Supplementary Figure S3), suggesting that future ocean warming may promote the growth of these bacteria and potentially stimulate degradation of organic matters with a possibility to indirectly affect MPn-based methane production by the diazotroph.

While the specific growth rates of *Trichodesmium* grown with the MPn as the main phosphorus source (Figure 1) were comparable to that of *Trichodesmium* grown on inorganic phosphate (Breitbarth et al., 2007; Jiang et al., 2018; Yi et al., 2020), *Trichodesmium* grown on MPn demonstrated resilience to temperature as low as 16°C, contrasting sharply with the constraints observed in *Trichodesmium* cultured with inorganic phosphate. Specifically, *Trichodesmium* IMS101 did not grow below 20°C when supplied with inorganic phosphate (Breitbarth et al., 2007). In studies involving three other strains of *Trichodesmium erythraeum* (KO4-20, RLI, 21–75), the minimum growth temperature was reported to be 18°C (Fu et al., 2014). In the oceans, *Trichodesmium* is predominately found in tropical and subtropical waters with temperatures exceeding 20°C (Laroche and Breitbarth, 2005; Luo et al., 2012). Although its presence in higher latitudes with colder waters is occasionally reported (Díez et al., 2012; Rees et al., 2016; Sabeur et al., 2016), it had been thought that these *Trichodesmium* cells were merely transported by ocean currents and were unable to establish a sustained presence (Laroche and Breitbarth, 2005). Our results provide an additional interpretation for *Trichodesmium*’s persistence in higher latitudes where temperatures drop below 20°C. When MPn was utilized as the primary phosphorus source, *Trichodesmium* demonstrated the capacity to acclimate and grow at temperatures as low as 16°C, possibly sustaining a seed community in colder regions (Shaika et al., 2023). However, the underlined mechanisms for the tolerance of the low temperature in *Trichodesmium* grown with Mpn need to be explored in future studies.

Based on the significant positive correlations between methane production and the assimilation of carbon, nitrogen, and phosphorus, as well as with the specific growth rate (μ) and relative electron transport rate (rETR) (Figures 4, 5), we established a series of methane production quotients (MPQ), representing the ratios between key metabolic rates and methane production rates (Table 2). Given that *Trichodesmium’s* capacity for carbon and nitrogen fixation has been extensively studied over the past few decades (Sañudo-Wilhelmy et al., 2001; Mulholland et al., 2006; Bergman et al., 2013), we applied the methane production quotients for carbon and nitrogen to estimate *Trichodesmium*’s counteractive roles in mitigating greenhouse effect. Based on the MPQ (Table 2), for every 227–494 nmol of CO_2_ assimilated by *Trichodesmium*, 1 nmol of CH_4_ is emitted. Given that the global warming potential of CH_4_ is about 80 times that of CO_2_ (Collins et al., 2013), and taking into account that the contribution of MPn accounts for 5–9.8% (Clark et al., 1998; Repeta et al., 2016; Sosa et al., 2020) of the other phosphorus sources in the oceanic areas where *Trichodesmium* thrives, the CH_4_ emission via MPn demethylation from *Trichodesmium* can offset 0.25–1.12% of its CO_2_ mitigation effects. This calculation does not take the oxidation of methane during diffusion processes into account (Hakemian and Rosenzweig, 2007). As *Trichodesmium* inhabit the upper 40 meters and form extensive sea surface blooms, it is plausible to assume that methane produced by *Trichodesmium* will be released to the atmosphere. Furthermore, methane production in *Trichodesmium* is mainly coupled with the utilization of MPn. Investigating the concentrations of MPn in different waters would promote more accurate estimates on a global scale.

Under the influence of global warming, concurrent ocean warming (Collins et al., 2013) is predicted to shoal thermal stratification within the upper layers of the oceans (Somavilla et al., 2017; Frischkorn et al., 2018; Ulloa et al., 2019) and reduce the upward transport of dissolved inorganic phosphate (DIP). Consequently, *Trichodesmium* may increasingly resort to utilizing MPn, potentially leading to enhanced methane production. Additional drivers may impact methane production. The optimal growth temperature changes in Fe-replete versus Fe-deplete cells (Jiang et al., 2018). The Fe and MPn availability may further affect methane production and requires future study.

## Data availability statement

The raw data supporting the conclusions of this article will be made available by the authors, without undue reservation.

## Author contributions

CZ: Writing – review & editing, Data curation, Formal analysis, Investigation, Methodology, Software, Validation, Visualization, Writing – original draft. XY: Visualization, Writing – review & editing. HL: Writing – review & editing, Investigation. MB: Writing – review & editing. IB-F: Writing – review & editing, Funding acquisition. KG: Writing – review & editing, Conceptualization, Funding acquisition, Project administration, Resources, Supervision.

## References

[ref1] Amstrup S. K. Ong S. C. Sofos N. Karlsen J. L. Skjerning R. B. Boesen T.. (2023). Structural remodelling of the carbon–phosphorus lyase machinery by a dual ABC ATPase. Nat. Commun. 14:1001. doi: 10.1038/s41467-023-36604-y, PMID: 36813778 PMC9947105

[ref2] Behrenfeld M. J. O’Malley R. T. Siegel D. A. McClain C. R. Sarmiento J. L. Feldman G. C.. (2006). Climate-driven trends in contemporary ocean productivity. Nature 444, 752–755. doi: 10.1038/nature05317, PMID: 17151666

[ref3] Bergman B. Sandh G. Lin S. Larsson J. Carpenter E. J. (2013). *Trichodesmium*-a widespread marine cyanobacterium with unusual nitrogen fixation properties. FEMS Microbiol. Rev. 37, 286–302. doi: 10.1111/j.1574-6976.2012.00352.x, PMID: 22928644 PMC3655545

[ref01] Berman-Frank I. Lundgren P. Chen Y. B. Kupper H. Kolber Z. Bergman B. Falkowski P. (2001). Segregation of nitrogen fixation and oxygenic photosynthesis in the marine cyanobacterium Trichodesmium. Sci. 294, 1534–1537. doi: 10.1126/science.1064082 11711677

[ref4] Beversdorf L. J. White A. E. Björkman K. M. Letelier R. M. Karl D. M. (2010). Phosphonate metabolism by *Trichodesmium* IMS101 and the production of greenhouse gases. Limnol. Oceanogr. 55, 1768–1778. doi: 10.4319/lo.2010.55.4.1768

[ref5] Bižić M. Ionescu D. Günthel M. Tang K. W. Grossart H.-P. (2018). “Oxic methane cycling: new evidence for methane formation in oxic lake water” in Biogenesis of hydrocarbons, 1–22.

[ref6] Bižić M. Klintzsch T. Ionescu D. Hindiyeh M. Günthel M. Muro-Pastor A. M.. (2020). Aquatic and terrestrial cyanobacteria produce methane. Sci. Adv. 6:eaax5343. doi: 10.1126/sciadv.aax5343, PMID: 31998836 PMC6962044

[ref7] Björkman K. M. Karl D. M. (2003). Bioavailability of dissolved organic phosphorus in the euphotic zone at station ALOHA, North Pacific subtropical gyre. Limnol. Oceanogr. 48, 1049–1057. doi: 10.4319/lo.2003.48.3.1049

[ref8] Boatman T. G. Lawson T. Geider R. J. (2017). A key marine Diazotroph in a changing ocean: the interacting effects of temperature, CO_2_ and light on the growth of *Trichodesmium erythraeum* IMS101. PLoS One 12:e0168796. doi: 10.1371/journal.pone.0168796, PMID: 28081236 PMC5230749

[ref9] Born D. A. Ulrich E. C. Ju K.-S. Peck S. C. Van Der Donk W. A. Drennan C. L. (2017). Structural basis for methylphosphonate biosynthesis. Science 358, 1336–1339. doi: 10.1126/science.aao3435, PMID: 29217579 PMC5901744

[ref10] Breitbarth E. Oschlies A. Laroche J. (2007). Physiological constraints on the global distribution of *Trichodesmium*–effect of temperature on diazotrophy. Biogeosciences 4, 53–61. doi: 10.5194/bg-4-53-2007

[ref11] Capone D. G. Zehr J. P. Paerl H. W. Bergman B. Carpenter E. J. (1997). Trichodesmium, a globally significant marine cyanobacterium. Science 276, 1221–1229. doi: 10.1126/science.276.5316.1221

[ref12] Chappell P. D. Webb E. A. (2010). A molecular assessment of the iron stress response in the two phylogenetic clades of *Trichodesmium*. Environ. Microbiol. 12, 13–27. doi: 10.1111/j.1462-2920.2009.02026.x, PMID: 19708870

[ref13] Chen Y.-B. Dominic B. Mellon M. T. Zehr J. P. (1998). Circadian rhythm of nitrogenase gene expression in the diazotrophic filamentous nonheterocystous cyanobacterium Trichodesmium sp. strain IMS 101. J. Bacteriol. 180, 3598–3605. doi: 10.1128/jb.180.14.3598-3605.1998, PMID: 9658003 PMC107328

[ref14] Chen Y. B. Zehr J. P. Mellon M. (1996). Growth and nitrogen fixation of the diazotrophic filamentous nonheterocystous cyanobacterium Trichodesmium sp. Ims 101 in defined media: evidence for a circadian rhythm. J. Phycol. 32, 916–923. doi: 10.1111/j.0022-3646.1996.00916.x

[ref15] Clark L. L. Ingall E. D. Benner R. (1998). Marine phosphorus is selectively remineralized. Nature 393:426. doi: 10.1038/30881

[ref16] Collins M. Knutti R. Arblaster J. Dufresne J.-L. Fichefet T. Friedlingstein P.. (2013). “Chapter 12 - Long-term climate change: Projections, commitments and irreversibility,” in Climate Change 2013: The Physical Science Basis. IPCC Working Group I Contribution to AR5. IPCC, Cambridge: Cambridge University Press.

[ref17] Damm E. Helmke E. Thoms S. Schauer U. Nöthig E. Bakker K.. (2010). Methane production in aerobic oligotrophic surface water in the Central Arctic Ocean. Biogeosciences 7, 1099–1108. doi: 10.5194/bg-7-1099-2010

[ref18] Díez B. Bergman B. Pedrós-Alió C. Antó M. Snoeijs P. (2012). High cyanobacterial nifH gene diversity in Arctic seawater and sea ice brine. Environ. Microbiol. Rep. 4, 360–366. doi: 10.1111/j.1758-2229.2012.00343.x, PMID: 23760800

[ref19] Dodd A. N. Salathia N. Hall A. Kévei E. Tóth R. Nagy F.. (2005). Plant circadian clocks increase photosynthesis, growth, survival, and competitive advantage. Science 309, 630–633. doi: 10.1126/science.1115581, PMID: 16040710

[ref20] Dyhrman S. T. (2016). “Nutrients and their acquisition: phosphorus physiology in microalgae” in The physiology of microalgae, 155–183.

[ref21] Dyhrman S. T. Chappell P. D. Haley S. T. Moffett J. W. Orchard E. D. Waterbury J. B.. (2006). Phosphonate utilization by the globally important marine diazotroph *Trichodesmium*. Nature 439, 68–71. doi: 10.1038/nature04203, PMID: 16397497

[ref22] Dyhrman S. T. Webb E. A. Anderson D. M. Moffett J. W. Waterbury J. B. (2002). Cell-specific detection of phosphorus stress in *Trichodesmium* from the Western North Atlantic. Limnol. Oceanogr. 47, 1832–1836. doi: 10.4319/lo.2002.47.6.1832

[ref23] Eilers P. Peeters J. (1988). A model for the relationship between light intensity and the rate of photosynthesis in phytoplankton. Ecol. Model. 42, 199–215. doi: 10.1016/0304-3800(88)90057-9

[ref24] Frischkorn K. R. Krupke A. Guieu C. Louis J. Rouco M. Salazar Estrada A. E.. (2018). Trichodesmium physiological ecology and phosphate reduction in the western tropical South Pacific. Biogeosciences 15, 5761–5778. doi: 10.5194/bg-15-5761-2018

[ref25] Fu F.-X. Yu E. Garcia N. S. Gale J. Luo Y. Webb E. A.. (2014). Differing responses of marine N_2_ fixers to warming and consequences for future diazotroph community structure. Aquat. Microb. Ecol. 72, 33–46. doi: 10.3354/ame01683

[ref26] Genty B. Briantais J.-M. Baker N. R. (1989). The relationship between the quantum yield of photosynthetic electron transport and quenching of chlorophyll fluorescence. Biochim. Biophys. Acta 990, 87–92. doi: 10.1016/S0304-4165(89)80016-9

[ref27] Günthel M. Klawonn I. Woodhouse J. Bižić M. Ionescu D. Ganzert L.. (2020). Photosynthesis-driven methane production in oxic lake water as an important contributor to methane emission. Limnol. Oceanogr. 65, 2853–2865. doi: 10.1002/lno.11557

[ref28] Hakemian A. S. Rosenzweig A. C. (2007). The biochemistry of methane oxidation. Annu. Rev. Biochem. 76, 223–241. doi: 10.1146/annurev.biochem.76.061505.175355 17328677

[ref29] Haydon M. J. Mielczarek O. Robertson F. C. Hubbard K. E. Webb A. A. (2013). Photosynthetic entrainment of the *Arabidopsis thaliana* circadian clock. Nature 502, 689–692. doi: 10.1038/nature12603, PMID: 24153186 PMC3827739

[ref30] IPCC (2013). Climate Change 2013: The Physical Science Basis. Contribution of Working Group I to the Fifth Assessment Report of the Intergovernmental Panel on Climate Change eds. Stocker, T. F, D. Qin, G.-K. Plattner, M. Tignor, S.K. Allen, J. Boschung, et al. (Cambridge, United Kingdom and New York, NY, USA: Cambridge University Press), 1535.

[ref31] Jiang H.-B. Fu F.-X. Rivero-Calle S. Levine N. M. Sañudo-Wilhelmy S. A. Qu P.-P.. (2018). Ocean warming alleviates iron limitation of marine nitrogen fixation. Nat. Clim. Chang. 8, 709–712. doi: 10.1038/s41558-018-0216-8

[ref32] Johnson K. M. Hughes J. E. Donaghay P. L. Sieburth J. M. (1990). Bottle-calibration static head space method for the determination of methane dissolved in seawater. Anal. Chem. 62, 2408–2412. doi: 10.1021/AC00220A030

[ref33] Kamat S. S. Williams H. J. Raushel F. M. (2011). Intermediates in the transformation of phosphonates to phosphate by bacteria. Nature 480, 570–573. doi: 10.1038/nature10622, PMID: 22089136 PMC3245791

[ref34] Karl D. M. Beversdorf L. Björkman K. M. Church M. J. Martinez A. Delong E. F. (2008). Aerobic production of methane in the sea. Nat. Geosci. 1, 473–478. doi: 10.1038/ngeo234

[ref35] Karl D. M. Björkman K. M. (2015). Dynamics of dissolved organic phosphorus. in Biogeochemistry of marine dissolved organic matter eds. D. A. Hansell and C. A. Carlson (Elsevier), 233–334.

[ref36] Karl D. M. Tilbrook B. D. (1994). Production and transport of methane in oceanic particulate organic matter. Nature 368, 732–734. doi: 10.1038/368732a0

[ref37] Klintzsch T. Geisinger H. Wieland A. Langer G. Nehrke G. Bizic M.. (2023). Stable carbon isotope signature of methane released from phytoplankton. Geophys. Res. Lett. 50:e2023GL103317. doi: 10.1029/2023GL103317

[ref38] Klintzsch T. Langer G. Nehrke G. Wieland A. Lenhart K. Keppler F. (2019). Methane production by three widespread marine phytoplankton species: release rates, precursor compounds, and potential relevance for the environment. Biogeosciences 16, 4129–4144. doi: 10.5194/bg-16-4129-2019

[ref39] Klintzsch T. Langer G. Wieland A. Geisinger H. Lenhart K. Nehrke G.. (2020). Effects of temperature and light on methane production of widespread marine phytoplankton. *Journal of geophysical research*. Biogeosciences 125:e2020JG005793. doi: 10.1029/2020JG005793

[ref40] Kolowith L. C. Ingall E. D. Benner R. (2001). Composition and cycling of marine organic phosphorus. Limnol. Oceanogr. 46, 309–320. doi: 10.4319/lo.2001.46.2.0309

[ref41] Lamontagne R. Swinnerton J. Linnenbom V. Smith W. (1973). Methane concentrations in various marine environments. J. Geophys. Res. 78, 5317–5324. doi: 10.1029/JC078i024p05317

[ref42] Laroche J. Breitbarth E. (2005). Importance of the diazotrophs as a source of new nitrogen in the ocean. J. Sea Res. 53, 67–91. doi: 10.1016/j.seares.2004.05.005

[ref43] Lenhart K. Klintzsch T. Langer G. Nehrke G. Bunge M. Schnell S.. (2016). Evidence for methane production by the marine algae Emiliania huxleyi. Biogeosciences 13, 3163–3174. doi: 10.5194/bg-13-3163-2016

[ref44] Liu L. Y. Xie G. J. Ding J. Liu B. F. Xing D. F. Ren N. Q.. (2022). Microbial methane emissions from the non-methanogenesis processes: a critical review. Sci. Total Environ. 806:151362. doi: 10.1016/j.scitotenv.2021.151362, PMID: 34740653

[ref45] Luo Y.-W. Doney S. Anderson L. Benavides M. Berman-Frank I. Bode A.. (2012). Database of diazotrophs in global ocean: abundance, biomass and nitrogen fixation rates. Earth Syst. Sci. Data 4, 47–73. doi: 10.5194/essd-4-47-2012

[ref46] Mao S.-H. Zhang H.-H. Zhuang G.-C. Li X.-J. Liu Q. Zhou Z.. (2022). Aerobic oxidation of methane significantly reduces global diffusive methane emissions from shallow marine waters. Nat. Commun. 13:7309. doi: 10.1038/s41467-022-35082-y, PMID: 36437260 PMC9701681

[ref47] Martinez A. Ventouras L. A. Wilson S. T. Karl D. M. Delong E. F. (2013). Metatranscriptomic and functional metagenomic analysis of methylphosphonate utilization by marine bacteria. Front. Microbiol. 4:340. doi: 10.3389/fmicb.2013.00340, PMID: 24324460 PMC3840354

[ref48] Masotti I. Ruiz-Pino D. Le Bouteiller A. (2007). Photosynthetic characteristics of Trichodesmium in the Southwest Pacific Ocean: importance and significance. Mar. Ecol. Prog. Ser. 338, 47–59. doi: 10.3354/meps338047

[ref49] Mccarthy J. J. Carpenter E. J. (1979). Oscillatoria *Trichodesmium thiebautii* (cyanophyta) in the central North Atlantic Ocean. J. Phycol. 15, 75–82. doi: 10.1111/j.1529-8817.1979.tb02965.x

[ref50] Metcalf W. W. Griffin B. M. Cicchillo R. M. Gao J. Janga S. C. Cooke H. A.. (2012). Synthesis of methylphosphonic acid by marine microbes: a source for methane in the aerobic ocean. Science 337, 1104–1107. doi: 10.1126/science.1219875, PMID: 22936780 PMC3466329

[ref02] Milligan A. J. Berman-Frank I. Gerchman Y. Dismukes G. C. Falkowski P. G. (2007). Light-dependent oxygen consumption in nitrogen-fixing cyanobacteria plays a key role in nitrogenase protection. J. Psychol. 43, 845–852. doi: 10.1111/j.1529-8817.2007.00395.x

[ref51] Mulholland M. R. Bernhardt P. W. (2005). The effect of growth rate, phosphorus concentration, and temperature on N2 fixation, carbon fixation, and nitrogen release in continuous cultures of Trichodesmium IMS101. Limnol. Oceanogr. 50, 839–849. doi: 10.4319/lo.2005.50.3.0839

[ref52] Mulholland M. R. Bernhardt P. W. Heil C. A. Bronk D. A. O'Neil J. M. (2006). Nitrogen fixation and release of fixed nitrogen by *Trichodesmium spp*. in the Gulf of Mexico. Limnol. Oceanogr. 51, 1762–1776. doi: 10.4319/lo.2006.51.4.1762

[ref53] Norberg J. (2004). Biodiversity and ecosystem functioning: a complex adaptive systems approach. Limnol. Oceanogr. 49, 1269–1277. doi: 10.4319/lo.2004.49.4_part_2.1269

[ref54] Ohki K. Zehr J. P. Fujita Y. (1992). Regulation of nitrogenase activity in relation to the light-dark regime in the filamentous non-heterocystous cyanobacterium *Trichodesmium sp*. NIBB 1067. Microbiology 138, 2679–2685. doi: 10.1099/00221287-138-12-2679

[ref55] Padfield D. Yvon-Durocher G. Buckling A. Jennings S. Yvon-Durocher G. (2016). Rapid evolution of metabolic traits explains thermal adaptation in phytoplankton. Ecol. Lett. 19, 133–142. doi: 10.1111/ele.12545, PMID: 26610058 PMC4991271

[ref56] Polovina J. J. Howell E. A. Abecassis M. (2008). Ocean's least productive waters are expanding. Geophys. Res. Lett. 35:L03618. doi: 10.1029/2007GL031745

[ref57] Ralph P. J. Gademann R. (2005). Rapid light curves: a powerful tool to assess photosynthetic activity. Aquat. Bot. 82, 222–237. doi: 10.1016/j.aquabot.2005.02.006

[ref58] Rees A. P. Tait K. Widdicombe C. E. Quartly G. D. Mcevoy A. J. Al-Moosawi L. (2016). Metabolically active, non-nitrogen fixing, *Trichodesmium* in UK coastal waters during winter. J. Plankton Res. 38, 673–678. doi: 10.1093/plankt/fbv123, PMID: 27274100 PMC4892227

[ref59] Repeta D. J. Ferrón S. Sosa O. A. Johnson C. G. Repeta L. D. Acker M.. (2016). Marine methane paradox explained by bacterial degradation of dissolved organic matter. Nat. Geosci. 9, 884–887. doi: 10.1038/ngeo2837

[ref60] Ritchie R. J. (2006). Consistent sets of spectrophotometric chlorophyll equations for acetone, methanol and ethanol solvents. Photosynth. Res. 89, 27–41. doi: 10.1007/s11120-006-9065-9, PMID: 16763878

[ref61] Sabeur H. I. Wafa F.-S. Asma H. Malika B. H. (2016). Long term characterization of *Trichodesmium erythraeum* blooms in Gabès gulf (Tunisia). Cont. Shelf Res. 124, 95–103. doi: 10.1016/j.csr.2016.05.007

[ref62] Sañudo-Wilhelmy S. A. Kustka A. B. Gobler C. J. Hutchins D. A. Yang M. Lwiza K.. (2001). Phosphorus limitation of nitrogen fixation by *Trichodesmium* in the Central Atlantic Ocean. Nature 411, 66–69. doi: 10.1038/35075041, PMID: 11333977

[ref63] Scranton M. I. Brewer P. G. (1977). Occurrence of methane in the near-surface waters of the western subtropical North-Atlantic. Deep-Sea Res. 24, 127–138. doi: 10.1016/0146-6291(77)90548-3

[ref64] Shaika N. A. Alhomaidi E. Sarker M. M. An Nur A. Sadat M. A. Awal S.. (2023). Winter bloom of marine cyanobacterium, Trichodesmium erythraeum and its relation to environmental factors. Sustain. For. 15:1311. doi: 10.3390/su15021311

[ref65] Sohm J. A. Capone D. G. (2006). Phosphorus dynamics of the tropical and subtropical North Atlantic: Trichodesmium spp. versus bulk plankton. Mar. Ecol. Prog. Ser. 317, 21–28. doi: 10.3354/meps317021

[ref66] Solórzano L. Sharp J. H. (1980). Determination of total dissolved phosphorus and particulate phosphorus in natural waters. Limnol. Oceanogr. 25, 754–758. doi: 10.4319/lo.1980.25.4.0754

[ref67] Somavilla R. González-Pola C. Fernández-Diaz J. (2017). The warmer the ocean surface, the shallower the mixed layer. How much of this is true? J. Geophys. Res. Oceans 122, 7698–7716. doi: 10.1002/2017JC013125, PMID: 29201584 PMC5699439

[ref68] Sosa O. A. Burrell T. J. Wilson S. T. Foreman R. K. Karl D. M. Repeta D. J. (2020). Phosphonate cycling supports methane and ethylene supersaturation in the phosphate-depleted western North Atlantic Ocean. Limnol. Oceanogr. 65, 2443–2459. doi: 10.1002/lno.11463

[ref69] Stasi R. Neves H. I. Spira B. (2019). Phosphate uptake by the phosphonate transport system PhnCDE. BMC Microbiol. 19:79. doi: 10.1186/s12866-019-1445-3, PMID: 30991951 PMC6469041

[ref70] Stihl A. Sommer U. Post A. F. (2001). Alkaline phosphatase activities among populations of the colony-forming diazotrophic cyanobacterium Trichodesmium spp.(cyanobacteria) in the Red Sea. J. Phycol. 37, 310–317. doi: 10.1046/j.1529-8817.2001.037002310.x

[ref71] Taenzer L. Carini P. C. Masterson A. M. Bourque B. Gaube J. H. Leavitt W. D. (2020). Microbial methane from methylphosphonate isotopically records source. Geophys. Res. Lett. 47:e2019GL085872. doi: 10.1029/2019gl085872

[ref72] Tong S. Hutchins D. A. Gao K. (2019). Physiological and biochemical responses of *Emiliania huxleyi* to ocean acidification and warming are modulated by UV radiation. Biogeosciences 16, 561–572. doi: 10.5194/bg-16-561-2019

[ref73] Ulloa H. N. Winters K. B. Wüest A. Bouffard D. (2019). Differential heating drives downslope flows that accelerate mixed-layer warming in ice-covered waters. Geophys. Res. Lett. 46, 13872–13882. doi: 10.1029/2019GL085258

[ref74] Van't Hoff J. H. Lehfeldt R. A. (1899). Lectures on Theoretical and Physical Chemistry. Nature 59:557. doi: 10.1038/059557b0

[ref75] Von Arx J. N. Kidane A. T. Philippi M. Mohr W. Lavik G. Schorn S.. (2023). Methylphosphonate-driven methane formation and its link to primary production in the oligotrophic North Atlantic. Nat. Commun. 14:6529. doi: 10.1038/s41467-023-42304-4, PMID: 37845220 PMC10579326

[ref76] Wang X. (2022). Physiological and elemental changes of Trichodesmium in response to growth limitation by phosphorus, iron and zinc. PhD thesis, Christian-Albrechts-Universität.

[ref77] Wang Q. Alowaifeer A. Kerner P. Balasubramanian N. Patterson A. Christian W.. (2021). Aerobic bacterial methane synthesis. Proc. Natl. Acad. Sci. USA 118:e2019229118. doi: 10.1073/pnas.2019229118, PMID: 34183407 PMC8271786

[ref78] Weber T. Wiseman N. A. Kock A. (2019). Global Ocean methane emissions dominated by shallow coastal waters. Nat. Commun. 10:4584. doi: 10.1038/s41467-019-12541-7, PMID: 31594924 PMC6783430

[ref79] Yi X. Fu F.-X. Hutchins D. A. Gao K. (2020). Light availability modulates the effects of warming in a marine N_2_ fixer. Biogeosciences 17, 1169–1180. doi: 10.5194/bg-17-1169-2020

[ref80] Yvon-Durocher G. Caffrey J. M. Cescatti A. Dossena M. Giorgio P. D. Gasol J. M.. (2012). Reconciling the temperature dependence of respiration across timescales and ecosystem types. Nature 487, 472–476. doi: 10.1038/nature11205, PMID: 22722862

[ref81] Zehr J. P. Capone D. G. (2020). Changing perspectives in marine nitrogen fixation. Science 368:eaay9514. doi: 10.1126/science.aay9514, PMID: 32409447

