# Supplementary material for: Correlation of methane production with physiological traits in Trichodesmium IMS 101 grown with methylphosphonate at different temperatures
Source: Front Microbiol. 2024 Jun 4;15:1396369. doi: 10.3389/fmicb.2024.1396369 (PMC11184136; doi:10.3389/fmicb.2024.1396369)
Supplement: Supplementary file 1 [file Table_1.docx]

Supplementary Materials for

Correlation of methane production with physiological traits in *Trichodesmium IMS101* grown with methylphosphonate at different temperatures

Chuze Zou^1^, Xiangqi Yi^2^, He Li^3^, Mina Bizic^4^, Ilana Berman-Frank^5^, and Kunshan Gao^1,3*^

^1^State Key Laboratory of Marine Environmental Science, College of the Ocean and Earth Sciences, Xiamen University, Xiamen, China

^2^Polar and Marine Research Institute, College of Harbor and Coastal Engineering, Jimei University, Xiamen, China

^3^Co-Innovation Center of Jiangsu Marine Bio-industry Technology, Jiangsu Ocean

University, Lianyungang 222005, China.

^4^Department of Environmental Microbiomics, Technical University of Berlin, Berlin, Germany.

^5^Department of Marine Biology, Leon H. Charney School of Marine Sciences, University of Haifa, Haifa, Israel.

* Corresponding author: Kunshan Gao ([ksgao@xmu.edu.cn](mailto:ksgao@xmu.edu.cn))

## Supplementary Figures


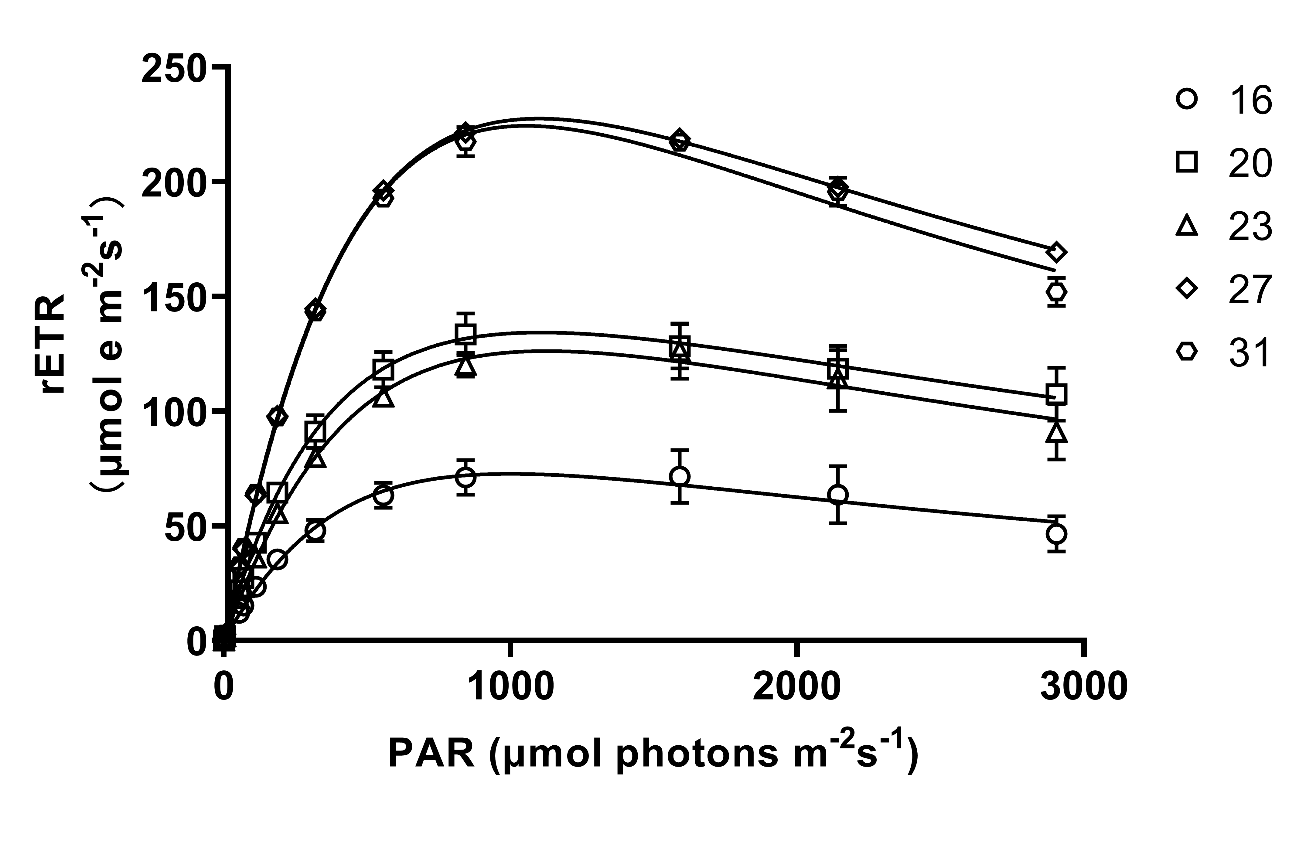


**Supplementary Figure 1.** Rapid light curves of *Trichodesmium* IMS101 after cultures were acclimated for 6 months to the specific temperatures ranging from 16 to 31℃. The symbols represent the growth-acclimated temperatures (℃). Values represent the means ± SD of measurements for independent biological replicates (n = 3).


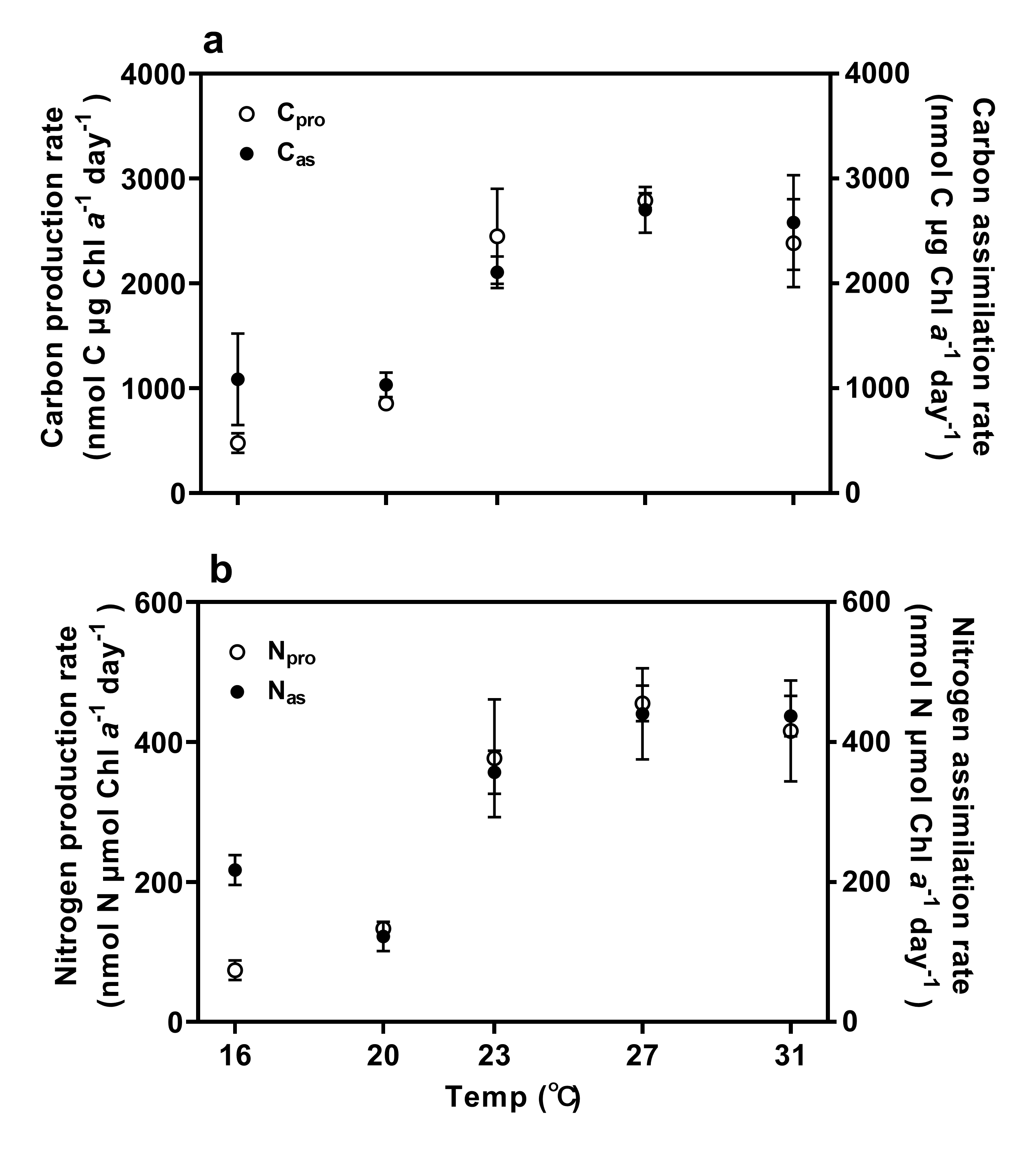


**Supplementary Figure 2** Carbon assimilation rates (a) and nitrogen assimilation rate (b) were calculated as POC or PON content  × specific growth rate (white circle) and estimated based on POC or PON changes (black circle) of *Trichodesmium* IMS101 after cultures were acclimated for 6 months to the specific temperatures ranging from 16 to 31℃. Values represent means ± SD of independent biological replicates (n = 3).


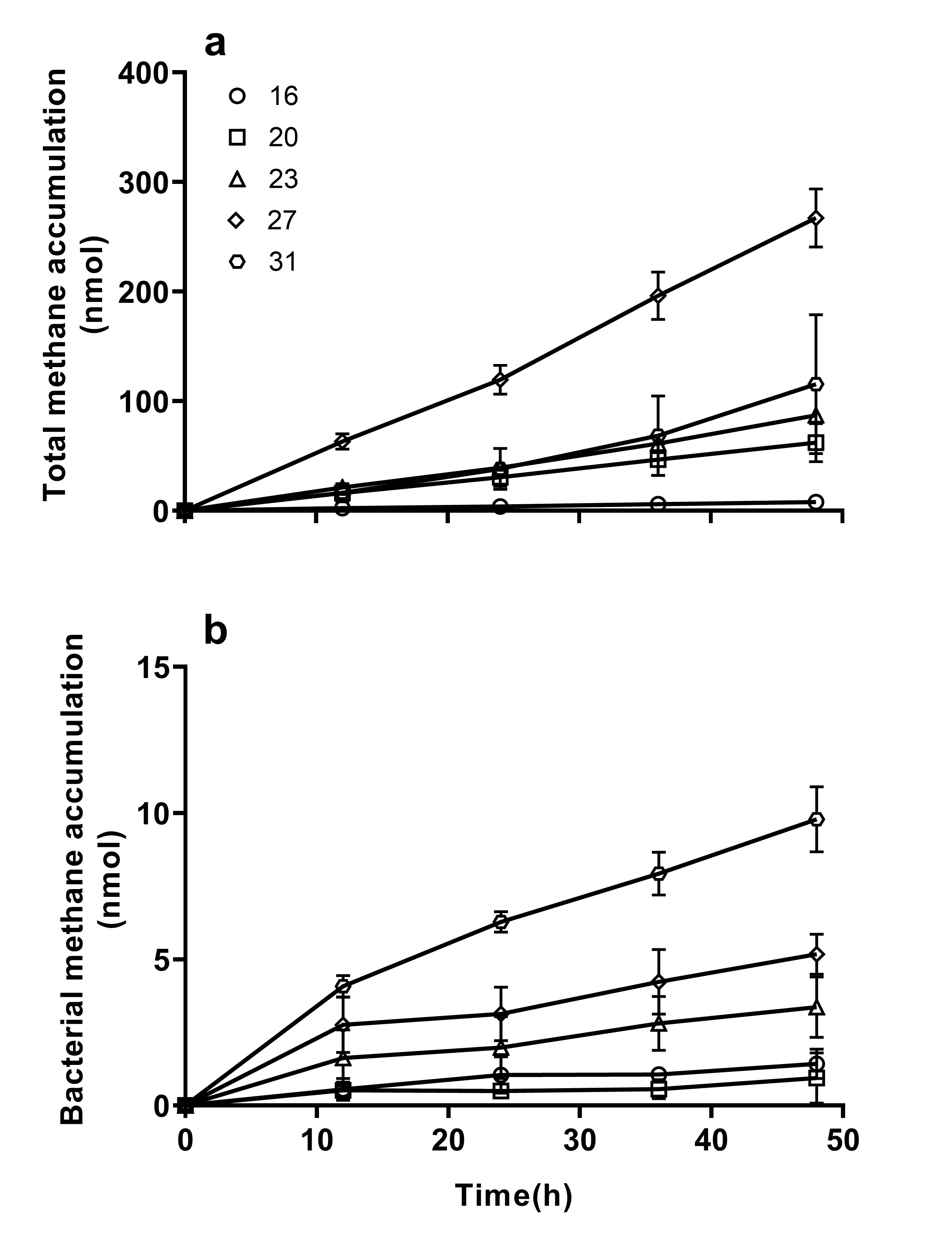


**Supplementary Figure 3** Total methane accumulation of *Trichodesmium* IMS101 cultures (a) and bacterial controls without *Trichodesmium* (b) after having acclimated for 6 months to the temperatures ranging from 16 to 31℃. The numbers next to the symbols indicate the growth temperatures (℃). Values are the means ± SD of independent biological replicates (n = 3).


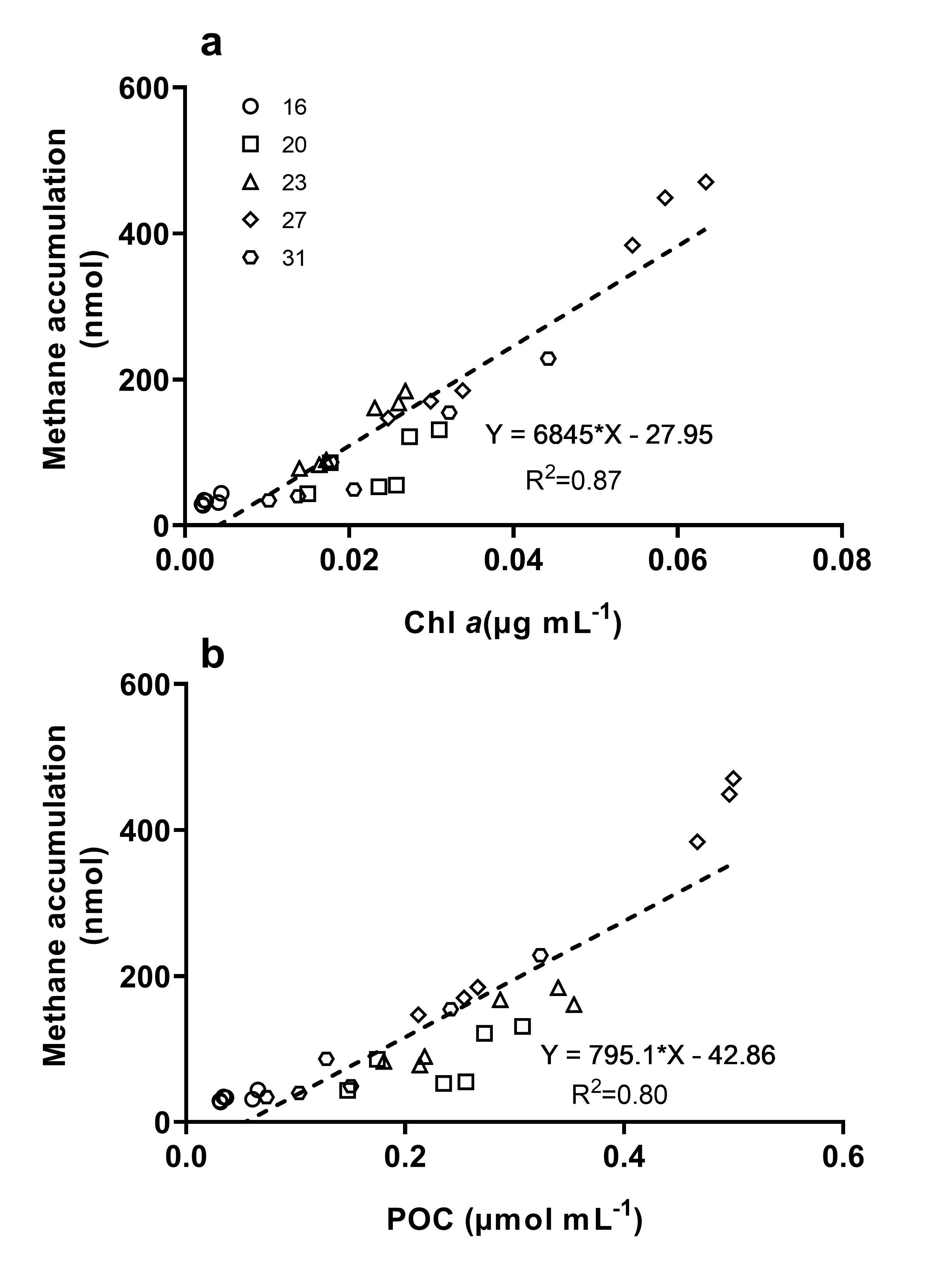


**Supplementary Figure 4.** The correlation of total methane accumulation with biomass in *Trichodesmium* IMS101: (a) Chl a and (b) Particulate Organic Carbon (POC) after the cells having acclimated for 6 months to the temperatures ranging from 16 to 31℃. The numbers next to the symbols denote the growth temperatures (℃). Each data point represents an independent biological replicate, and different symbols represent different biological replicates.

**Supplementary Table 1** Temperature coefficients (Q_10_) of growth, carbon, nitrogen, phosphorus assimilation and methane production and the activation energy (E_a_) of carbon, nitrogen, phosphorus assimilation and methane production in *Trichodesmium* IMS101 after having acclimated for 6 months to the temperatures ranged from 16 to 31℃. "NA" denotes not applied. The values represent the means ± SD of independent biological replicates (n = 3).

| Parameters | Q_10_ | E_a_ (eV) |
| --- | --- | --- |
| μ | 8.6±2.3 | NA |
| C_as_ | 4.9±0.7 | 1.19 ± 0.19 |
| N_as_ | 2.6±0.5 | 0.78 ± 0.25 eV |
| P_as_ | 2.6±0.3 | NA |
| CH_4pro_ | 4.6±0.7 | 1.08 ± 0.08 |
